# Inhibition of SphK1 reduces radiation-induced migration and enhances sensitivity to cetuximab treatment by affecting the EGFR/SphK1 crosstalk

**DOI:** 10.18632/oncotarget.2436

**Published:** 2014-09-10

**Authors:** Carlotta Schiefler, Guido Piontek, Johannes Doescher, Dominik Schuettler, Martin Mißlbeck, Martina Rudelius, Anna Haug, Rudolf Reiter, Gero Brockhoff, Anja Pickhard

**Affiliations:** ^1^ Department of Otolaryngology Head and Neck Surgery, Technical University of Munich, Ismaninger Str. 22, 81675 Muenchen, Germany; ^2^ Department of Radiotherapy, Technical University of Munich, Ismaninger Str. 22, 81675 Muenchen, Germany; ^3^ Institute of Pathology, Julius-Maximilians-University and Comprehensive Cancer Center Mainfranken, Josef-Schneider-Straße 2, 97080 Wuerzburg, Germany; ^4^ Department of Otolaryngology, University of Innsbruck, Anichstr. 35, 6020 Innsbruck, Austria; ^5^ Department of Otolaryngology Head and Neck Surgery, Section of Phoniatrics and Pedaudiology, University of Ulm, Prittwitzstr. 43, 89075 Ulm, Germany; ^6^ Department of Gynecology and Obstetrics; University of Regensburg, Landshuter Str. 65, 93053 Regensburg, Germany

**Keywords:** SphK1, EGFR, HNSCC, radiation, radiation-induced migration

## Abstract

SphK1 is known to play a role in tumor progression, resistance to radiochemotherapy, and migration patterns. As the overall survival rates of squamous cell carcinoma of the head and neck (HNSCC) remain poor due to limitations in surgery and irradiation and chemotherapy resistance, SphK1 is an important enzyme to investigate. The purpose of this study was to elucidate the impact of SphK1 on irradiation efficacy of HNSCC *in-vitro* with emphasis on EGFR signaling. By immunhistochemical staining we found a positive correlation between EGFR and SphK1 expression in patient specimens. In colony formation assays irradiation sensitive cell lines showed a poor response to cetuximab, an EGFR inhibitor, and SKI-II, a SphK1 inhibitor, and vice versa. In irradiation sensitive cells an enhanced reduction of cell migration and survival was found upon simultaneous targeting of EGFR and SphK1. In the present study, we elucidated a linkage between the two signaling pathways with regard to the efficacy of cetuximab treatment and the impact on the migration behavior of tumor cells. We investigated the biological impact of inhibiting these pathways and examined the biochemical implications after different treatments. An understanding of the processes involved could help to improve the treatment of patients with HNSCC.

## INTRODUCTION

Head and neck squamous cell carcinoma (HNSCC) is the sixth most common cancer worldwide [1]. Mortality due to this cancer remains largely unimproved despite ongoing advancements in tumor surgery and radio- and chemotherapy, with 5-year survival rates under 50% [2]. Prognoses of HNSCC disease are often difficult due to a poor control of loco-regional disease and significant morbidity [3].

An elevated expression of epidermal growth factor receptor (EGFR) is significantly associated with a reduced progression-free survival [4]. Indeed, EGFR overexpression and alteration has been attributed to HNSCC pathogenesis and progression. Hence, anti-EGFR treatment regimens for HNSCC have been implemented [5]. Mechanistically, EGFR targeting with the human-murine chimeric immunoglobulin G_1_ cetuximab competitively prevents receptor activation by endogenous ligands and results in EGFR downreguation by internalization of the receptor/antibody complex [6]. We previously described an irradiation induced migration of HNSCC cell lines that could be linked to EGFR overexpression and could be reversed by blocking the EGFR activity with the EGFR kinase inhibitor AG1478 [7, 8].

Another prognostic indicator for HNSCC is sphingosine kinase 1 (SphK1), a lipid kinase that catalyzes the conversion of sphingosine to sphingosine-1-phosphate (S1P), a biologically active lipid that plays an important role in mammalian cell growth, survival, and migration [9]. It has been shown that SphK1 mRNA expression is significantly elevated in several malignant diseases e.g., of the breast, colon, uterus, lung, ovary, kidney, and rectum [10]. Increased levels of S1P result from elevated SphK1 expression/activity and were associated with suppression of apoptosis [11], enhanced cell growth and migration [12], augmented angiogenesis [13], pronounced resistance to irradiation [14], and chemotherapy [15, 16]. Similarly, Sinah et al. found a correlation between the tumor stage and elevated SphK1 expression in HNSCC [17].

Receptor crosstalk between members of different receptor families is typical for transversal signal transduction over the cell membrane [10]. The activation mechanisms of SphK1 often involves a variety of growth factors, amongst them e.g., platelet-derived growth factor (PDGF), EGF, nerve growth factor (NGF), insulin growth factor (IGF), and transforming growth factor beta (TGFβ) [10]. Naturally, SphK1/S1P tyrosin kinase receptor/RTK (EGFR) receptor interaction results in a shared intracellular signaling network. Hobson et al. found that cell migration stimulated by PDGF, which stimulates sphingosine kinase and increases intracellular S1P, was dependent on the expression of S1P-1-receptor [18]. Shida et al. demonstrated that S1P induces the rapid and transient tyrosine phosphorylation of EGFR in gastric cancer cells [19]. However, studies on intermolecular receptor crosstalk in HNSCC are lacking and no information is available regarding the impact of anti-EGFR / SKI-II targeting, and concurrent irradiation.

In this study we inhibited the SphK1 using the non-competitive, kinase specific inhibitor 2-(p-hydroxyanilino)-4-(p-chlorophenyl)thiazole (SKI-II) that does not affect PKCα-, Erk2-, or PI3K-activity [20]. SKI-II also has the effect of reducing SphK1 protein expression [21]. This inhibitor reduces tumor growth in mice and shows a favorable *in-vivo* half-life period of 15.3 h [22]. We analyzed EGFR signaling, cell survival, and migration as a function of SphK1 targeting in HNSCC cell lines.

## RESULTS

### SphK1 is overexpressed in HNSCC compared to normal tissue

Immunhistochemical stainings was done on tumor samples of 180 patients. Table 1 shows the clinical data of these patients. Immunohistochemistry revealed that both proteins, EGFR (p < 0.001) and SphK1 (p < 0.01), were significantly higher expressed in the tumor samples compared to the non-cancerous tissue (Suppl. Fig. 1).

**Table 1:** Clinical characteristics of patients included in the study

Patient characteristics	Data
**age**	
average age/range	69/45–87 years
**sex**	
male/female	165/15
**pT classification**	
pT1/pT2/pT3/pT4	25/66/48/41
**pN classification**	
pN0/pN+	94/86
**cM classification**	
cM0/cM1	180/0
**grading**	
G1/G2/G3	10/110/60
**anatomic localisation**	
oral cavitiy/oropharynx/hypopharynx/larynx	33/58/33/56
**risk factor tobacco**	
smoker/non-smoker/not known	109/40/31
**risk factor alcohol**	
regularly/not regularly/not known	92/37/51

SphK1 and EGFR expression does not correlate with the clinical parameters staging, nodal status, metastasis, grading relapse and late metastasis rate. Only the lymph node stage and SphK1 overexpression were significantly correlated (p = 0.024) (Tab. 2). Tumors without a positive lymph node involvement more frequently overexpressed SphK1 than those with lymph node metastases. In addition, there was a highly significant correlation between SphK1 and EGFR expression (p = 0.01).

**Table 2:** p-values using Pearson's Chi-square Test. Cut-off score for samples considered having positive overexpression was 2

Marker	T	N	G	Relapse	Metastases
**EGFR**	0.220	0.195	0.069	0.886	0.365
**SphK1**	0.293	0.024	0.516	0.062	0.588

### HNCCC cell lines show different sensitivity to irradiation

**Figure 1:**
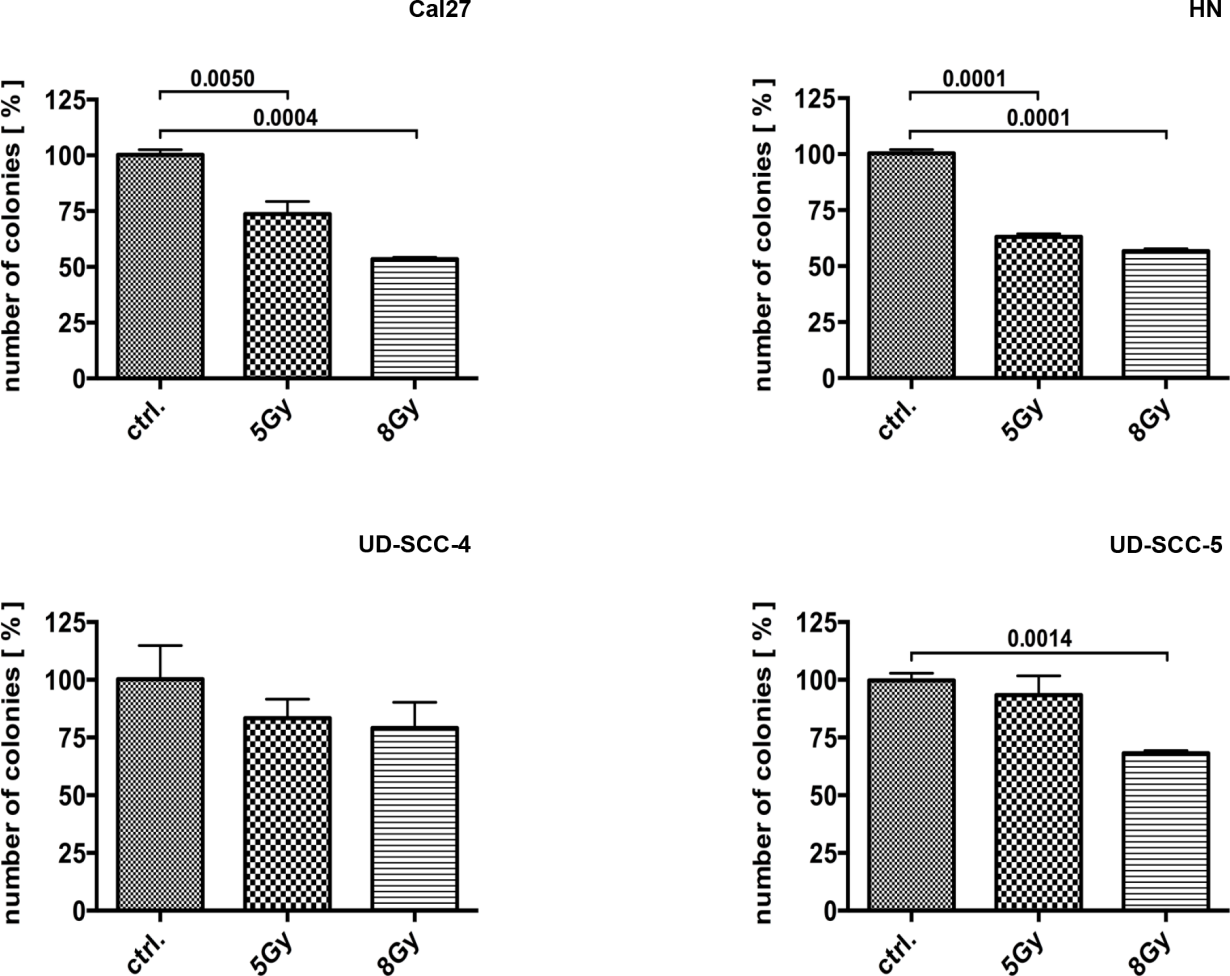
Colony formation assay performed on Cal27, HN, UD-SCC-4, and UD-SCC-5 cell lines as indicated: The cell lines showed different response to irradiation. Cal27 und HN cells appeared sensitive to irradiation whereas UD-SCC-4 and UD-SCC-5 cells were not. Irradiation-resistant UD-SCC-4 and UD-SCC-5 cell lines showed reduced clonogenic survival upon SKI-II and cetuximab treatment. In contrast, the clonogenic survival of irradiation sensitive Cal27 and HN cells is basically not reduced by SphK-1 and EGFR targeting with SKI-II and cetuximab.

The colony formation assay (CFA) revealed a strong impact of irradiation on Cal27 and HN cells, with almost 50% of the clones not surviving treatment doses of 8 Gy (Cal27, p = 0.0004; HN, p < 0.0001). In contrast, the UD-SCC-4 and UD-SCC-5 cells behaved largely insensitive to irradiation: The clonogenic survival of UD-SCC-4 was not significantly reduced by 5 or 8 Gy treatment doses. UD-SCC-5 survival was significantly reduced only upon irradiation with 8 Gy (p = 0.0014) (Fig. 1) (Suppl. Fig. 2).

### Radiation-resistant cell lines are sensitive to SKI-II and cetuximab therapy

**Figure 2:**
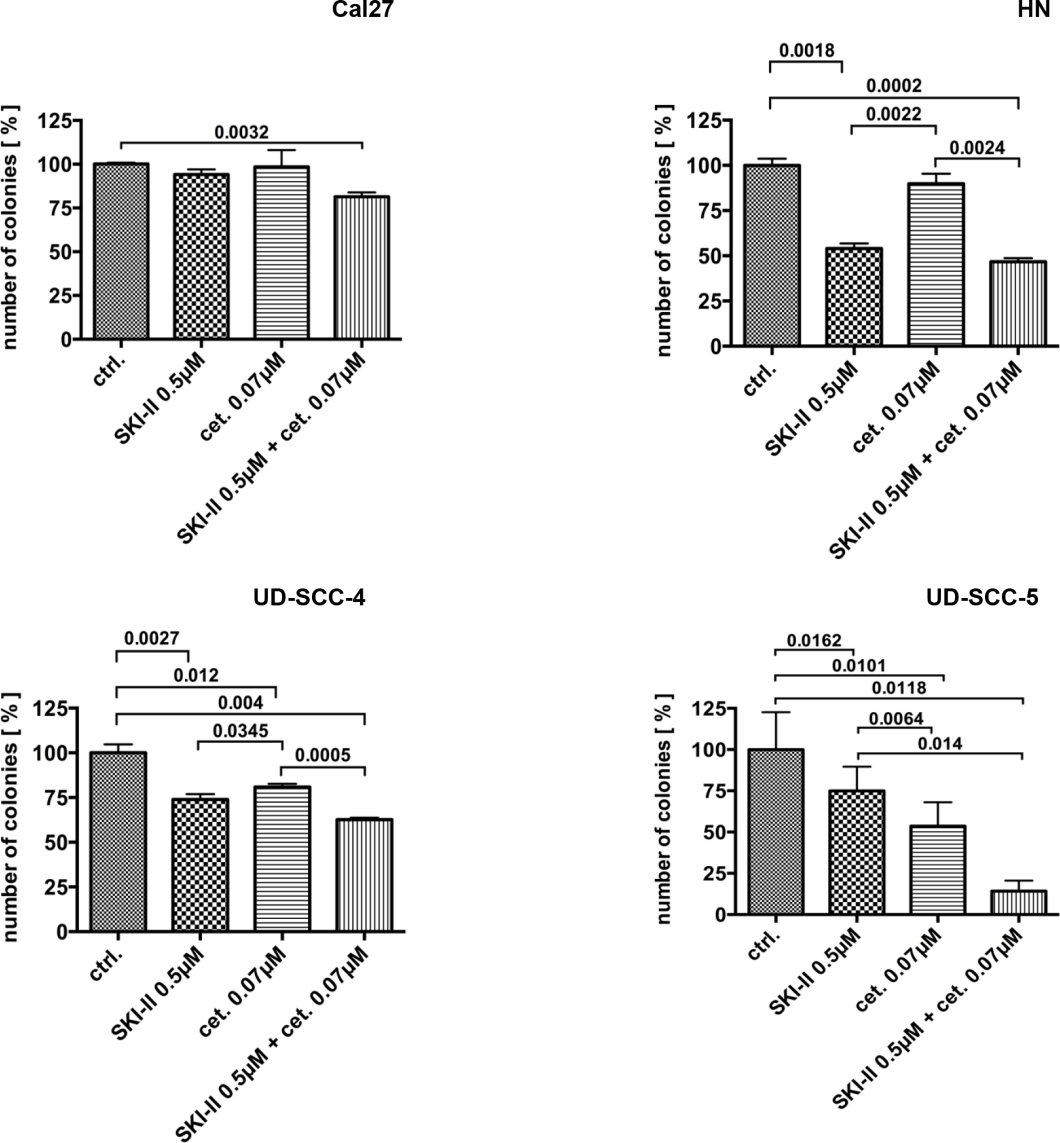
Colony formation assay performed on the four cell lines: Only combination treatment with SKI-II and cetuximab caused reduced clonogenic survival in all four cell lines.

Although the clonogenic survival of UD-SCC-4 and UD-SCC-5 cells was not severely affected by irradiation the outgrowth of these cell lines was significantly inhibited upon treatment with SKI-II and cetuximab in a dose-related manner (Fig. 2). Conversely, the clonogenic formation of the irradiation sensitive Cal27 and HN cells was not impaired upon treatment with SKI-II. The simultaneous administration of both SKI-II and cetuximab significantly reduced the clonogenic survival of UD-SCC-4, UD-SCC-5 and HN but not Cal27 cells (Fig. 2).

### Irradiation-sensitive cells show radiation-induced migration

**Figure 3:**
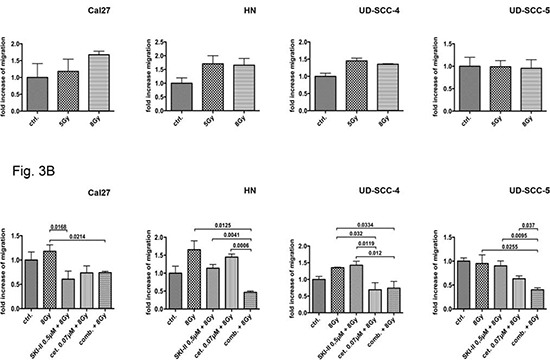
Wound healing assay: The radiation-sensitive cell line Cal27 reacted with a dose-dependent increase in migration after irradiation; in contrast, the resistant cell line UD-SCC-5 exhibited no radiation-induced migration. The HN and UD-SCC-4 cell lines shows a slightly increase of migration. The application of SKI-II and the combined administration of SKI-II and cetuximab inhibited Cal27 cell radiation-induced migration. The combination of inhibitors did not lead to an advantage compared to the single SKI-II treatment. Conversely, the single treatment of the inhibitors showed little impact on UD-SCC-5 cell migration patterns. Their combination, however, led to a significant reduction in migration, hence showing a significant advantage compared to the single treatment according to the CFA results.

Irradiation caused a dose dependent increase of migration activity in Cal27 whereas UD-SCC-5 cell migration was not affected by irradiation treatment. HN and UD-SCC-4 cell migration was stimulated by irradiation with 5 Gy. Irradiation with 8 Gy did not further enhance this effect (Fig. 3 A).

### SKI-II inhibits radiation-induced migration

The irradiation-induced migration of Cal27 cells was reduced by the simultaneous cell treatment with 0.5 μM SKI-II (p = 0.0168) but only a trend was seen after treatment with cetuximab (0.07 μM). Moreover, the combination of both inhibitors did not lead to a further inhibition than that of the SKI-II single treatment. Accordingly, in HN, UD-SCC-4, and UD-SCC-5 cells the combination treatment was most efficient (Fig. 3 B).

### E-cadherin expression is inversely correlated with irradiation-induced migration activity

**Figure 4:**
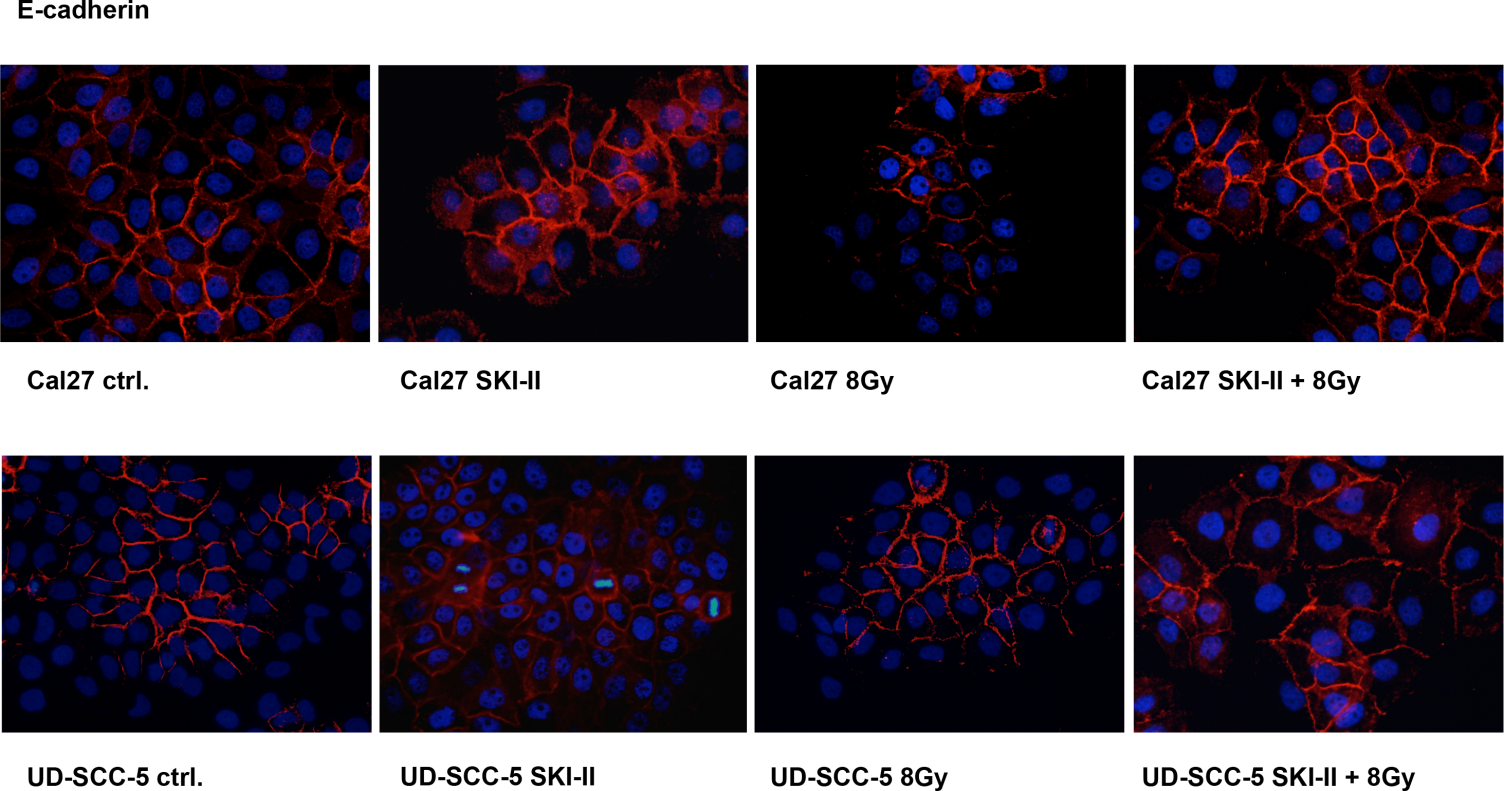
Immunofluorescence staining: Radiation doses of 8 Gy led to the degradation of E-cadherin in Cal27 cells; this effect, however, was obviated after pretreatment with SKI-II. Irradiation did not affect E-cadherin in UD-SCC-5 cells. SKI-II improved cell adhesion by upregulating the E-cadherin protein.

Immunofluorescence revealed reduced E-cadherin expression in Cal27 cells upon irradiation. E-cadherin expression in UD-SCC-5 cells was, however, unaltered upon pretreatment with SKI-II (Fig. 4).

### SphK1 is upregulation in cetuximab treated UD-SCC-5 cells

Due to the analogue response rates of Cal27 and HN cells on the one hand and the UD-SCC-4 and UD-SCC-5 cells on the other hand, migration assays were narrowed down to Cal27 and UD-SCC-5.

**Figure 5:**
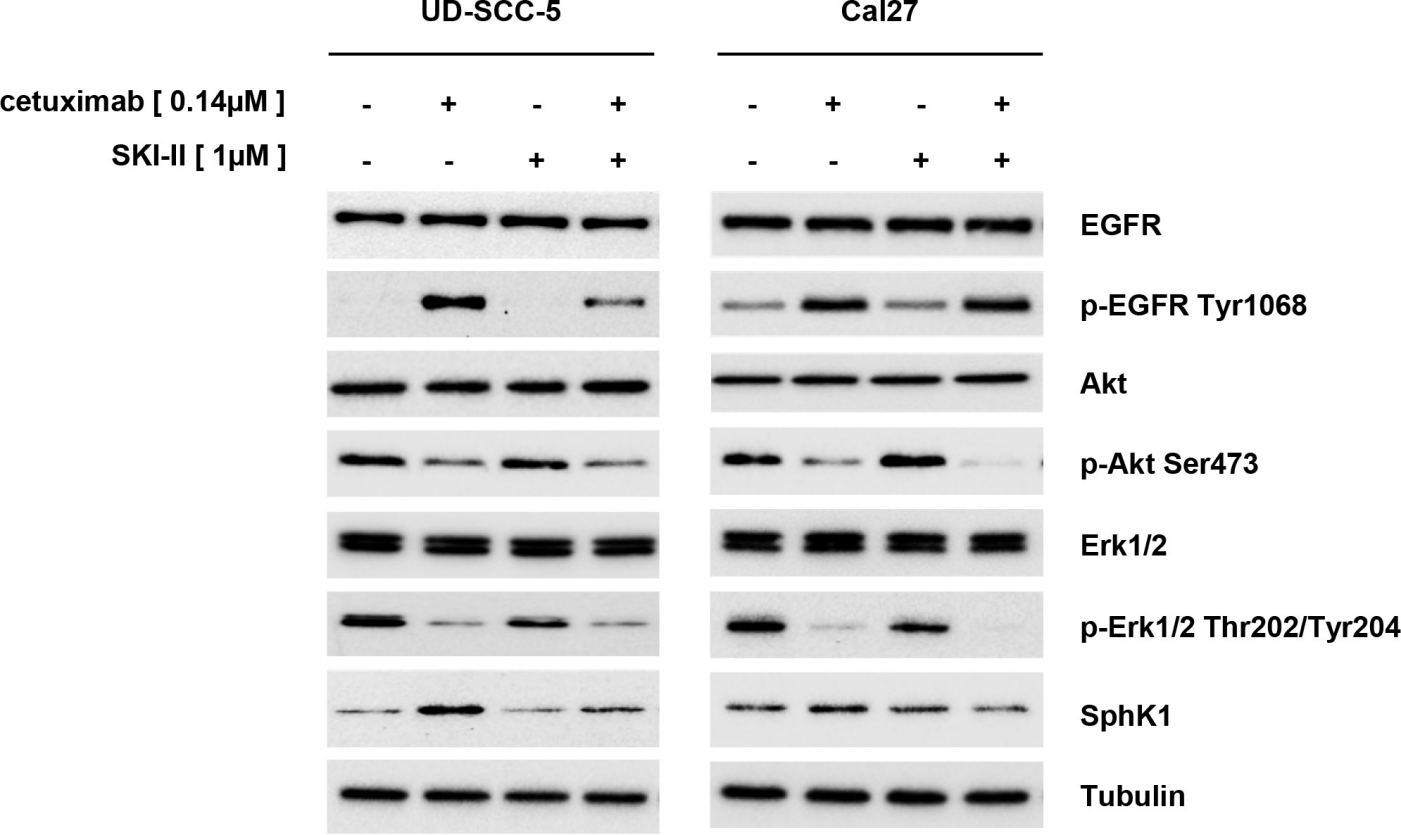
Western blot analyses: Cetuximab caused an increase in EGFR phosphorylation, which was not transmitted to the downstream Erk1/2 and Akt signaling pathways. Cetuximab also caused an upregulation of SphK1 protein in UD-SCC-5 cells but not in Cal27 cells. In UD-SCC-5 cells, however, the effect was attenuated by the addition of SKI-II.

EGFR targeting with cetuximab caused an increased receptor phosphorylation both in Cal27 and UD-SCC-5 cells. Nevertheless, the cetuximab treatment resulted in a reduced Akt and Erk1/2 phosphorylation. Strikingly, the SphK1 protein expression was upregulated upon cetuximab treatment in UD-SCC-5 but not in Cal27 cells (Fig. 5).

SKI-II treatment did not alter the SphK1 expression and did apparently not affect the Akt activity Cal27 and UD-SCC-5 cells. In contrast the SKI-II mediated inhibition of SphK1 considerably attenuated Erk1/2 phosphorylation at Thr202/Tyr204 residues.

The combined administration of SKI-II and cetuximab caused effects that were analogue to single cetuximab treatment although an upregulation of the SphK1 protein in UD-SCC-5 cells could not be found under this treatment conditions.

## DISCUSSION

We aimed to elucidate the role of SphK1 in HNSCC and the potential interactions with the EGFR signaling pathway. The protein expression of SphK1 and EGFR in the tumor tissues from 180 patients was analyzed and compared to the expression in non-cancerous tissues. Four human tumor cell lines were analyzed for their proliferation and survival capacity upon anti-SphK1 targeting with the highly specific SKI-II inhibitor and anti-EGFR targeting with cetuximab. In addition the treatment dependent migration proficiency was further analyzed. We used the SKI-II inhibitor although it is classified as carcinogenic / teratogenic. Blagosklonny, for example, outlined by a number of essays, that the carcinogenic/teratogenic effect of the substrate is expected from any useful anti-cancer drug [23–26].

According to other data [27–29] we found an elevated SphK1 and EGFR expression in HNSCC tissues compared to non-neoplastic tissues. In addition, we report for the first time a significant positive correlation of SphK1 and EGFR expression. This finding suggests a functional link of SphK1 and EGFR.

CFAs of irradiated and SKI-II / cetuximab treated cells revealed two groups of HNSCC cell lines: On the one hand UD-SCC-4 and UD-SCC-5 cells manifested as irradiation resistant but cetuximab and SKI-II sensitive. Treatment with the SphK-1 inhibitor SKI-II even restored irradiation sensitivity in these cells (data not shown), which has formerly been described elsewhere [17]. On the other hand HN and Cal27 cells were sensitive to irradiation but were resistant to SKI-II and cetuximab treatment. These findings suggest that it might be possible to stratify HNSCC patients into potential responders to either irradiation or target specific anti-SphK-1/EGFR treatment (Fig. 2, Suppl. Fig. 2).

**Figure 6:**
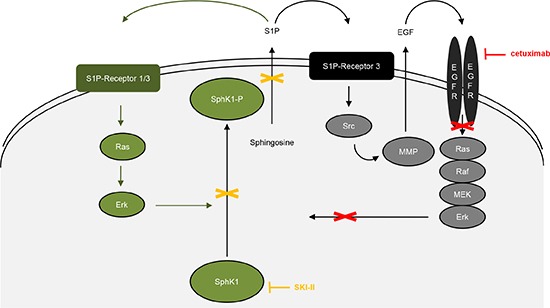
Model of EGFR/SphK1 interaction: SphK1 is a component of different amplification loops. One loop involves the transactivation of EGFR by S1P, causing the activation of Erk1/2 and thus the activation of SphK1. The other loop involves the activation of S1P receptors 1 and 3. Downstream signaling causes the activation of Erk1/2 via Ras and Erk1/2 is known to activate SphK1. Integrating these two amplification loops into one model leads to a new understanding of EGFR- and SphK1-targeted therapies, as treatment targeting EGFR only inhibits one loop. By western blot analyses, we found a compensatory upregulation of SphK1 after cetuximab treatment, leading to attenuated effects of the drug, as observed by CFA and WHA. However, blocking both loops and thus inhibiting the upregulation of the SphK1 protein leads to improved results of target therapies.

The combination treatment with cetuximab and SKI-II exceptionally reduced clonogenic survival of the inhibitor-sensitive UD-SCC-4 and UD-SCC-5 cells. The inhibition was even more pronounced at reduced doses compared to the effects caused by single treatments at higher doses (data not shown). The fact that cetuximab treatment caused an upregulation of SphK1 expression in UD-SCC-5 cells strongly indicates a functional link between EGFR and SphK1: It is known that S1P can transactivate several RTKs, including EGFR [10] and that the transactivation of EGFR involves S1P-3, Src, and MMP [19]. It is also known that EGFR downstream signaling leads to the activation of Erk1/2 [30] and that Erk1/2 activates SphK1 [31] (Fig. 6, illustration in black/gray). Furthermore, the downstream signaling of S1P receptors 1 and 3 causes activation of Erk1/2 [32] and consequently results in an activation of SphK1 [31] (Fig. 6, illustration in green). SphK1 is located at the critical point of a complex amplification loop, and this loop can apparently be broken through the combined treatment of cetuximab and SKI-II (Fig. 6).

Cell migration is essential for metastasis. Previous studies revealed a connection between SphK1 overexpression and migration capacity [12]. Here we show that SphK-1 and EGFR targeting with SKI-II and cetuximab inhibits the migration ability of HNSCC cell lines. Consistent with the reduced capacity to form cell colonies, we found an additive effect in UD-SCC-5 but not in Cal27 cells when both inhibitors were applied at the same time. Again a functional link between the SphK1 and EGFR signaling must be assumed.

Irradiation is an established treatment strategy for HNSCC patients. We previously reported radiation-induced migration in HNSCC cell lines [8]. The underlying mechanisms are, however, barely understood. Here we demonstrate that radiation-induced migration is more pronounced in cell lines that are sensitive to irradiation than in resistant cell lines. The irradiation enhanced migration capacity observed in Cal27 cells goes along with a downregulation of E-cadherin. Accordingly, the inhibition of SphK-1 by SKI-II treatment results in an upregulation of E-cadherin and simultaneously reduces the radiation-induced migration of Cal27 cells. The combination treatment with both inhibitors significantly affects the wound healing of UD-SCC-5 but not of Cal27. Thus, SphK1 and EGFR most likely affect the radiation-induced migration independently.

Cetuximab treatment of Cal27 and UD-SCC-5 cells caused EGFR phosphorylation at Tyr1068 but reduces the phosphorylation of Erk1/2 and Akt (Fig. 5). This finding is in agreement with data published elsewhere [33]. Even more strikingly, UD-SCC-5 cell treatment with cetuximab caused an upregulation of SphK1 protein that might abrogate the desired anti-tumorigenic effect. The SphK1 upregulation can, however, be repressed by the combined anti-EGFR / anti-SphK1 targeting that restores the sensitivity to either drug treatment.

In contrast to data published elsewhere [20] we found a decreased p-Erk1/2 Thr202/Tyr204 phosphorylation upon SKI-II treatment. This discrepancy can be explained by a reduced SphK1 product S1P upon treatment with SKI-II, because S1P can activate the S1P receptor and the downstream Erk1/2 signaling [32]. Moreover, S1P can transactivate RTKs (e.g. EGFR) and thus can cause Erk1/2 activation [10].

Overall, we provide strong evidence for a functional SphK1/EGFR interaction. To explain the effects described above, we developed the model illustrated in Fig. 6: Receptor specific targeting affects downstream signaling pathways of either receptor system and consequently tumor cell survival and migration. We suggest that cetuximab treatment, blocks just one of the associated signaling loops that results in a compensatory upregulation of the SphK1 protein (Fig. 6, green illustration). This in turn promotes the other signaling loop. Consequently, HNSCC treatment can be most efficiently improved by a synchronous targeting of both signaling loops i.e., by a combined anti SphK1/anti-EGFR treatment (Fig. 6, effecting green and black/gray loop). Our results indicate that the amplification loop is active in tumors that are sensitive to the inhibitors (e.g., represented by UD-SCC-5 cells) though resistance to inhibitors cannot be overcome by a combined treatment. Our results suggest the side effects of cetuximab treatment could possibly be ameliorated by a dose reduction.

## CONCLUSION

HNSCC cell lines (investigated in this study) can be graduated in 2 groups: the first is irradiation resistant but cetuximab and SKI-II sensitive, the second react conversely.

SKI-II was found to reduce malignancy in all cell lines, either directly by inhibiting proliferation, survival, and migration, or indirectly by inhibiting irradiation-induced migration. Also, a combination treatment with SKI-II and cetuximab could work efficiently in tumor cells which basically respond well to cetuximab treatment alone, because then lower cetuximab doses were required. However, possible side effects of SKI-II especially in combination treatments need to be further explored. The high effects achieved with the combination of cetuximab and SKI-II can most likely be attributed to positive signaling amplification loops between the EGFR- and SphK1 related pathways, although resistance to cetuximab or SKI-II could not be overcome by their combination. Therefore, we conclude that these amplification loops do not play a role in the development of resistance to cetuximab or SKI-II.

Currently, the therapy of patients with HNSCC in the advanced stage III and IV implies primary radiotherapy in combination with chemotherapy [34]. Our data indicate that a change in the therapeutic strategies of patients with HNSCC might be useful. Inhibition of the EGFR and SphK1 in combination with the radiotherapy might be an option to the conventional radiation and chemotherapy of patients with HNSCC.

## MATERIAL AND METHODS

### Tissue samples

Formalin-fixed and paraffin-embedded tumor samples from 180 patients with a squamous cell carcinoma of the oral cavity (n=33), oropharynx (n=58), hypopharynx (n=33), or larynx (n=56) were examined for EGFR and SphK1 expression. The expression levels were compared to non-cancerous tissue from the same patient.

The Medical Ethics Committee of the Technical University of Munich approved this study.

### Tissue microarray preparation

Core needle biopsies of viable, representative areas of each tumor specimen were retrieved from the original tumor blocks using a manual array (Beecher Instruments, Sun Prairie, Wisconsin, USA) and placed in a recipient paraffin array block. The goal was to obtain at least three tissue cylinders, with a diameter of 0.6 mm, from each biopsy specimen.

### Immunohistochemical staining

Fresh 1.5 μm sections from TMA blocks were transferred to glass slides, deparaffinized, and rehydrated. An antigen retrieval method (microwave oven heating in citrate-buffered saline) was applied following the instructions provided by the manufacturer. The TMA slides were cooled and incubated with antibodies against EGFR (rabbit, clone D38B1) 1:200 (Cell Signaling Technology, Danvers, USA) and SPHK1 (rabbit) 1:200 (Cell Signaling Technology, Danvers, USA). The reaction was developed using the labeled streptavidin-biotin-peroxidase system; DAB was used as the indicator. After counterstaining with hematoxylin, the slides were dehydrated in ascending concentrations of ethanol and mounted. Tissue with known expression of the respective antigen was used for a positive control; irrelevant antibodies of the immunoglobulin isotype were used as negative control.

### Scoring system for protein expression

A scoring system was used to describe the expression levels of the different proteins. The staining intensity was graded from 0 to 3 points (0 points = no staining, 1 point = low staining intensity, 2 points = moderate staining intensity, 3 points = strong staining intensity). The proportion of stained cells was estimated and graded from 0 to 4 points (0 points = 0% of the tumor cells, 1 points = <10% of the tumor cells, 2 points = 10%-29% of the tumor cells, 3 points = 30%-59% of the tumor cells, 4 points = 60%-100% of the tumor cells). Both scores were added to produce a cumulative score.

### Cell culture

The Cal27 and HN cell lines were obtained from DSMZ (Braunschweig, Germany), and UD-SCC-4 and UD-SCC-5 cells were obtained from the University of Düsseldorf (clinic for otolaryngology, Düsseldorf, Germany) (Tab. 3) [35]. The cells were cultured in Dulbecco's modified Eagle medium (DMEM) (Invitrogen, Darmstadt, Germany) containing 10% fetal calf serum (FBS) (Biochrom, Berlin Germany), 2 mM glutamine (Biochrom), 100 μg/ml streptomycin (Biochrom), and 100 U/ml penicillin (Biochrom), maintained at 37°C in an atmosphere of 5% CO_2_, and grown to 70–90% confluence.

**Table 3:** Data (HPV and p53 status raised in our lab) and clinical characterization of the cell lines used in the study

Cell line	Localisation	TNM	Grading	Age	Sex	HPV	p53
**Cal 27**	oral cavity	Tx Nx Mx	G3	56	male	negativ	Exon6 c.578a>t (H193L)
**HN**	oral cavity	Tx Nx M1	G2	60	male	negativ	Exon6 c.578a>t (H193L)
**UD-SCC-4**	oropharynx	T3 N1 M0	G2	45	male	negativ	Exon5 c.454c>g, c.557c>t, del. 460–472
**UD-SCC-5**	larynx	T1 N1 M0	G3	44	male	negativ	Exon5 c.535c>t (H179Y)

### Irradiation

Irradiation was performed at the Department of Radiotherapy (Technical University of Munich). The cells were X-irradiated with single doses of 5 or 8 Gy at room temperature using a Gulmay Medical X-ray source operated at 70 kV and a dose rate of approximately 1 Gy/min. The sham-treated group (0 Gy, control) was subjected to the same protocol as the exposed cells.

### Inhibitors

SphK1 was specifically inhibited using 2-(p-hydroxyanilino)-4-(p-chlorophenyl) thiazole (SKI-II) (Merck, Darmstadt, Germany) at concentrations of 0.5 μM and 1 μM. Cetuximab was purchased from Merck (Darmstadt, Germany) and used at concentrations of 0.07 μM and 0.14 μM.

### Colony formation assay

Cell survival was assessed by a colony formation assay (CFA). The cells were seeded in a 6-well plate (5 × 10^2^ cells/well). After 1 d, the cells were pretreated with cetuximab and SKI-II for one hour and subsequently irradiated; the cells were then cultured for 10 days. The cell colonies were formalin fixed and visualized by Crystal Violet staining (Aldrich Chemie, Steinheim, Germany); the colonies were counted after removing the dye by washing.

### Wound-healing assay

Cell migration was assessed by wound-healing assays (WHA). Cells were seeded in a 6-well plate (5 × 10^5^ cells/well), and a scratch was drawn into the cell layer after 2 d of incubation. The cells were then pretreated with inhibitors and optionally irradiated. Pictures of the scratch were obtained directly after and at 12 h after the treatment. The number of pixels covering with cells was evaluated using Photoshop, and the magnitude of the points of measurement was compared using the following formula:

((T2-T1) / 5038848) × 100

(T1 = 5038848 − the number of pixels at the start of measurement)

(T2 = 5038848 − the number of pixels after 12 h)

(5038848 = the total number of pixels)

### Immunofluorescence

For immunofluorescence (IF), slides were coated with 0.1% gelatin, and 2.5 × 10^5^ cells/slide were seeded. After 2 d of incubation, the cells were pretreated with inhibitors and irradiated. At 1 h after treatment, the cells were formalin fixed and stained with antibodies against the following proteins: E-cadherin (mouse) (Enzo Life Science, Lörrach, Germany), 1:200; EGFR (rabbit) (Santa Cruz Biotechnology, Dallas, Texas), 1:200; and SphK1 (rabbit) (Cell Signaling Technology, Danvers, USA), 1:200. Anti-rabbit-FITC (Cell Signaling Technology, Danvers, USA) and anti-mouse-Cyp3 (Cell Signaling Technology, Danvers, USA) were used for detection at a dilution of 1:200. The nuclei were stained with DAPI (1 μg/ml) before mounting cells with VectaShield® (Vector Laboratories, Burlingame, USA). Fluorescence images were captured with a Leica epifluorescence confocal microscope equipped with a digital camera and Leica Application Suite LAS V3.7 acquisition software.

### Western blotting

For protein isolation, the cells were starved under serum-free conditions for 12 h. One hour after treatment, the cells were lysed in 1x lysis buffer (New England Biolabs, Ipswich, USA) supplemented with 1 mM PMSF (Roth, Karlsruhe, Germany). Equal amounts of protein (15 μg) were separated by SDS-PAGE and transferred to Immobilon membranes (Millipore, Schwalbach, Germany). The blocking of nonspecific binding sites was performed using 5% (w/v) non-fat dry milk in TBST. The membranes were incubated with primary antibodies diluted in TBST for 12–14 hours at 4°C. HRP-conjugated immunoglobulins (diluted 1:5000 in 5% non-fat dry milk/TBST) served as detection antibodies and were probed for 1 h at room temperature. The immunoreactivity was visualized by exposure to high-performance chemoluminescence film (Biorad Laboratories, Munich, Germany). Antibodies against the following were used: p-EGFR Tyr1068 (Rabbit) (New England Biolabs) (1:2500); EGFR (Rabbit) (Santa Cruz Biotechnology, Dallas, Texas) (1:2500); p-Akt Ser473 (Rabbit) (New England Biolabs) (1:500); Akt (Rabbit) (New England Biolabs) (1:1000); p-Erk1/2 Thr202/Tyr204 (Rabbit) (New England Biolabs) (1:1000); Erk1/2 (Rabbit) (New England Biolabs) (1:1000); SphK1 (Rabbit) (New England Biolabs) (1:500); tubulin (Mouse) (Sigma Aldrich Chemie, Steinheim, Germany) (1:10000); anti-Rabbit HRP-linked IgG (Goat) (New England Biolabs) (1:5000); and anti-Mouse HRP linked IgG (Goat) (New England Biolabs) (1:2000).

### Statistical analysis

A statistical analysis of the protein expression in the tumor samples was performed using SPSS Statistics software (version 18/19, IBM, Munich, Germany). The relationship between the two proteins was tested by crosstabs and the Fisher exact test. Results with p-values <0.05 were considered significant. A statistical analysis for the *in vitro* experiments was performed using Prism Graph Pad 5.0 software. Assuming a symmetry correlation structure for all the experiments, all the hypotheses were tested with a one-way ANOVA. We compared the separate treatments and the untreated control for statistical significance with a t-test (p-values < 0.05).

## SUPPLEMENTARY FIGURES


