# Supplementary material for: Inhibition of SphK1 reduces radiation-induced migration and enhances sensitivity to cetuximab treatment by affecting the EGFR/SphK1 crosstalk
Source: Oncotarget. 2014 Sep 10;5(20):9877–88. doi: 10.18632/oncotarget.2436 (PMC4259444; doi:10.18632/oncotarget.2436)
Supplement: Supplementary file 1 [file oncotarget-05-9877-s001.pdf]

SUPPLEMENTARY FIGURES

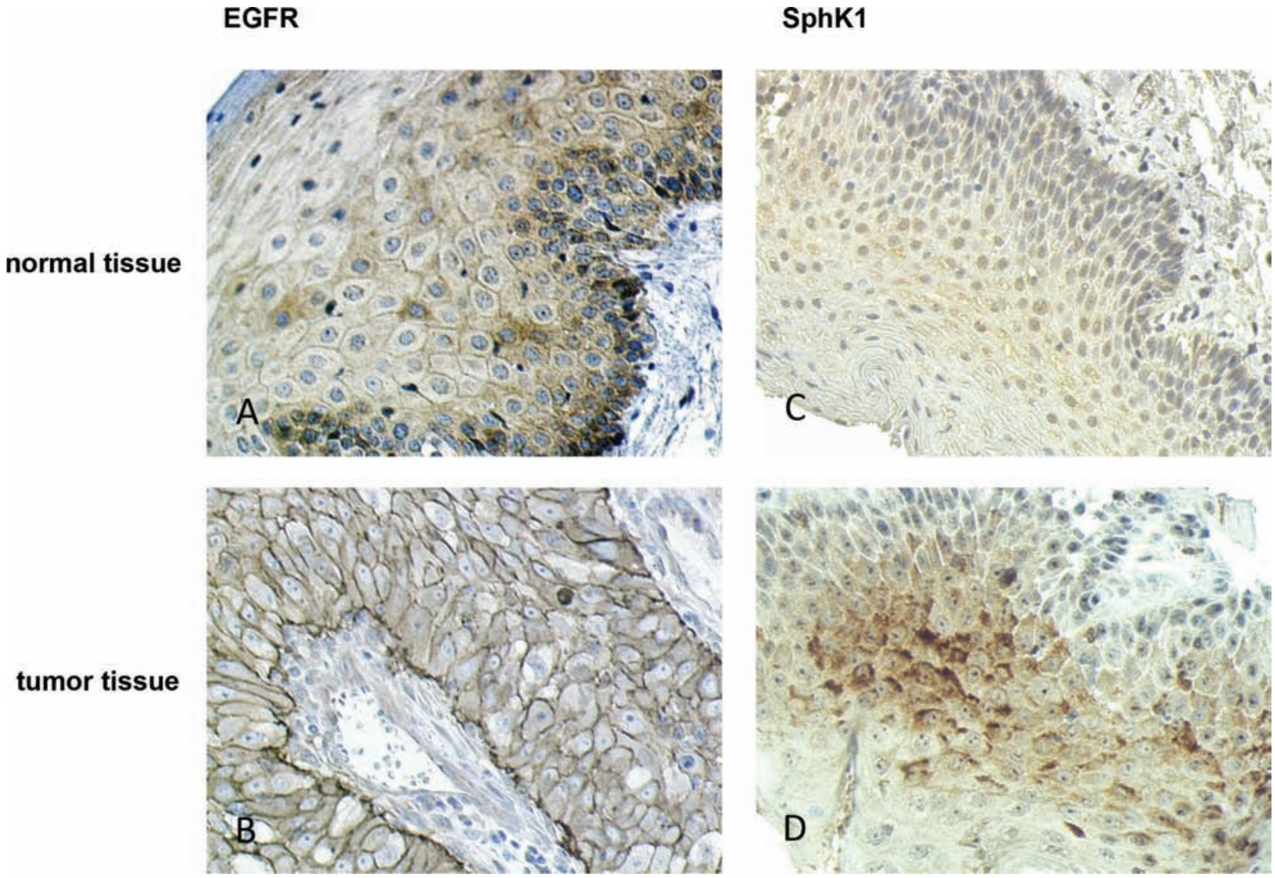

Supplementary Figure S1: Immunohistochemical staining: EGFR and SphK1 proteins were significantly higher expressed in tumors than in normal mucosa tissues. In addition, there was a significant positive correlation between the expression of SphK1 and EGFR proteins.

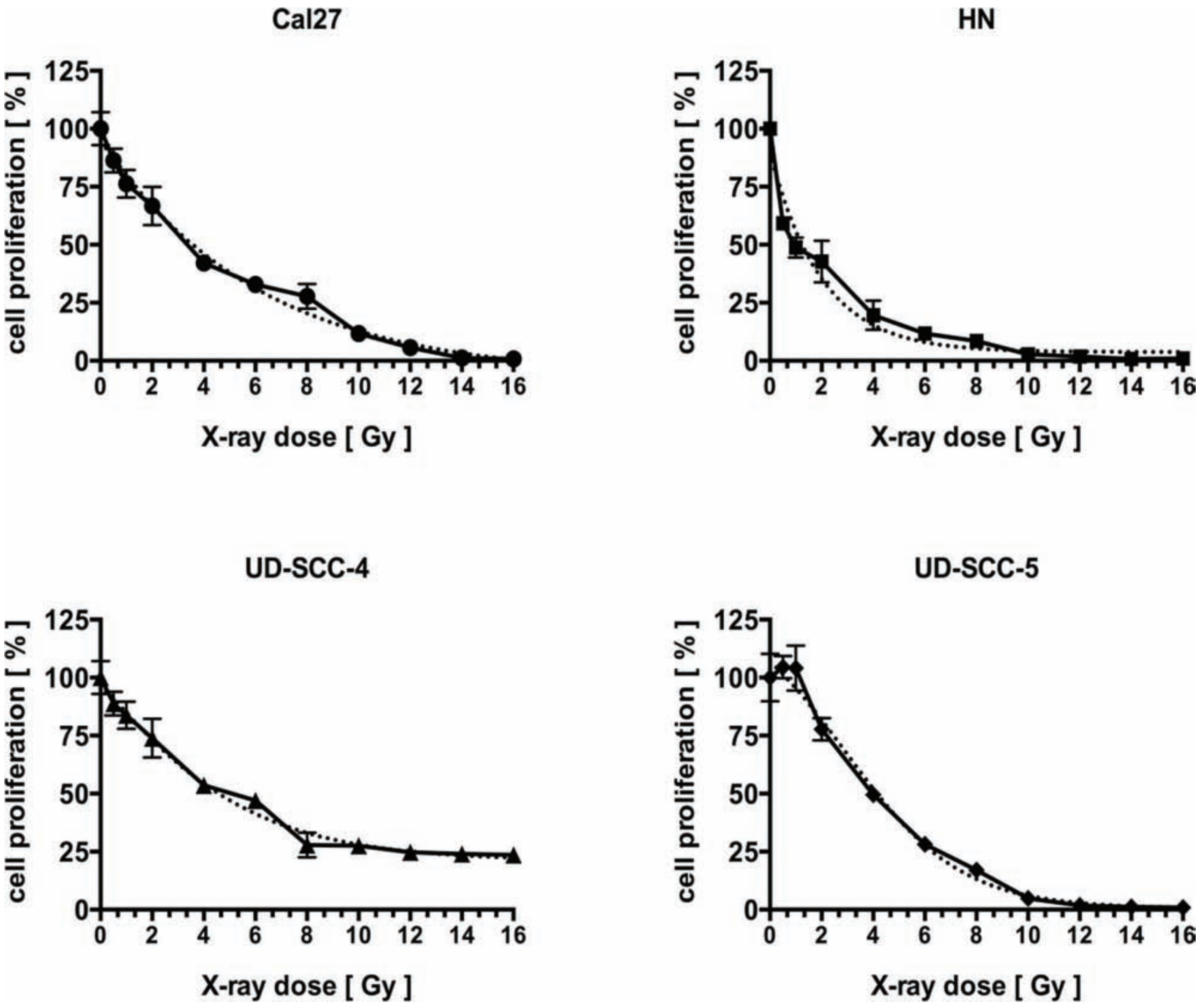

Supplementary Figure S2: Dose related curve of all four cell lines. We found a SF2 (Surviving Fraction at 2 Gy) of 50 to 60% in the radiosensitive cell lines Cal27 and HN. The radioresistant cell lines UD-SCC-4 and UD-SCC-5 shows 75% of cells alive.
